# Medical decision analysis for personalized oncology at the patient bedside

**DOI:** 10.1177/03008916251404283

**Published:** 2026-01-24

**Authors:** Paolo G. Casali, Lisa Licitra, Salvatore Provenzano, Ilaria Pellegrini, Chiara Fabbroni, Andrea Franza, Claudia Giani, Dario Callegaro, Stefano Cavalieri, Francesco Lanza, Anna Maria Frezza, Bruno Vincenzi, Renato Muzzini, Annalisa Trama, Rosalba Miceli, Gabriele Tinè, Valter Torri, Virginia Sanchini, Hykel Hosni, Paolo Bruzzi

**Affiliations:** 1 Department of Oncology and Hemato-oncology, University of Milan, Italy; 2 Mesenchymal and Rare Cancer Medical Oncology Unit, Fondazione IRCCS Istituto Nazionale dei Tumori, Milan, Italy; 3 Head and Neck Medical Oncology Department, Fondazione IRCCS Istituto Nazionale dei Tumori, Milan, Italy; 4 Academic Medical Oncology Unit, IRCCS Ospedale Policlinico San Martino, Genova, Italy; 5 Department of Medical Oncology, Fondazione IRCCS Istituto Nazionale dei Tumori, Milan, Italy; 6 Department of Surgery, Fondazione IRCCS Istituto Nazionale dei Tumori, Milan, Italy; 7 Ocular Oncology Unit, Department of Surgical Oncology, Fondazione IRCCS Istituto Nazionale dei Tumori di Milano, Italy; 8 Fondazione Policlinico Universitario Campus Bio-Medico, Rome, Italy; 9 Accademia Nazionale di Medicina, Genova, Italy; 10 Evaluative Epidemiology Unit, Fondazione IRCSS Istituto Nazionale dei Tumori, Milan, Italy; 11 Biostatistics for Clinical Research Unit, Fondazione IRCCS Istituto Nazionale dei Tumori, Milan, Italy; 12 Clinical Oncology Department, Istituto di Ricerche Farmacologiche Mario Negri IRCCS, Milan, Italy; 13 Centre for Biomedical Ethics and Law, Department of Public Health and Primary Care, KU Leuven, Leuven, Belgium; 14 Logic, Uncertainty, Computation and Information (LUCI) Lab, Department of Philosophy, University of Milan, Italy; 15 Independent Researcher Genova, Italy

**Keywords:** medical decision making, decision analysis, decision tree, treatment personalization, adjuvant treatment, cytoreduction, active surveillance, advanced cancer

## Abstract

Medical decision analysis is a method to make rational clinical decisions under uncertainty, enabling a mathematical combination of *probabilities* and *utilities* (i.e. values assigned to outcomes under risk). Decision analysis is commonly used in health economics, but it is underexploited in the clinic. With a view to fostering the use of medical decision analysis at the cancer patient bedside, this paper provides basic templates for some typical clinical decisions in cancer treatment, namely affecting: the quantity/quality of life trade-offs in curable cancer; adjuvant/neoadjuvant treatments; cytoreductive treatments; active surveillance / watchful waiting choices; treatment of advanced cancer; cancer follow-up. The clinical use of medical decision analysis is challenged by several difficulties, which are briefly recalled. Contrary to clinical research, medical decision analysis does not build new evidence: it simply provides physicians with a method to personalize clinical decisions on the basis of available evidence. Its added value in clinical practice correlates with the complexity of a decision. However, it also has a great potential in medical education, in order to empower clinicians with skills improving their ability to rationally shape medical decisions and share them properly with their patients.

## Introduction

Medical decision analysis (MDA) is a method to make rational clinical decisions under uncertainty, which is based on game and decision theory, as introduced in economics in the 1940s.^1^ The use of decision theory in medicine was proposed in 1959.^2^ Now, MDA relies on a large medical literature.^3-14^ Today, it is widely used in health economics to perform cost/effectiveness analyses.^3^ However, although its use in clinical practice has been encouraged by prestigious papers, handbooks, educational efforts, MDA has not reached the patient bedside. On the contrary, MDA could make clinical decision-making more rational, strengthen the practice of patient-physician shared decision-making and provide a method to the so-called “precision medicine”, or “personalized medicine”, at the heart of today’s healthcare agenda.^14^

This paper tries to provide some basic templates for typical clinical decisions in cancer treatment. Beforehand, MDA is briefly summarized.

### MDA in essence

MDA uses *decision trees* to conceptually structure and graphically represent medical decisions in such a way as to enable a mathematical combination of *probabilities* and *utilities*.^3-8^ A decision tree is built by a clinician facing a decision problem, however atypical and/or complex it may be. This stands at the opposite of so-called *clinical algorithms*, which are flow-charts representing state-of-the-art clinical sequences of decisions.^15^ In other words, clinical algorithms are pathways provided to the physician in order to follow them, while decision trees are conceived by the physician in order to formalize the clinical problem of the individual patient and solve it rationally.

**Figure 1.**
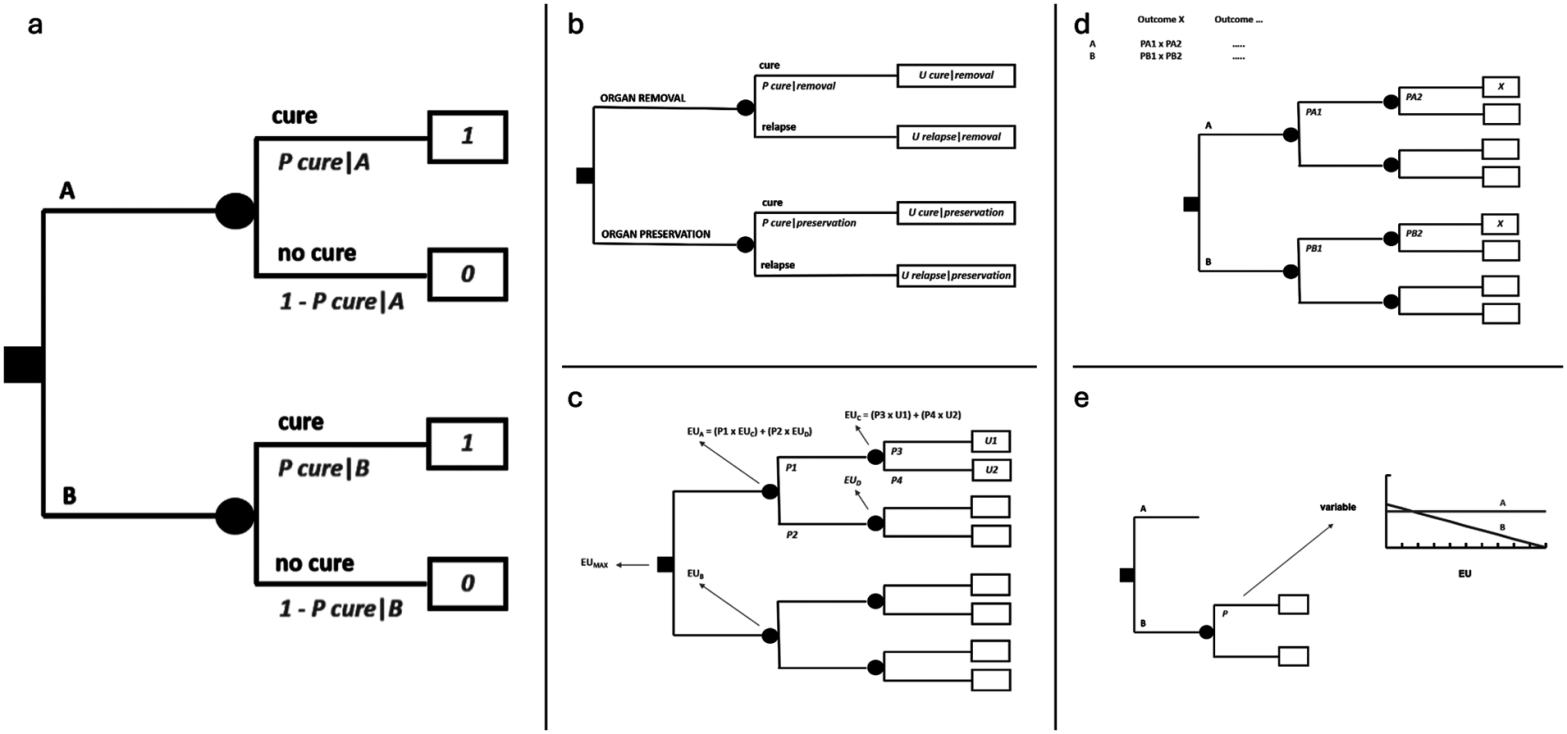
MDA in essence. (a) Basic decision tree. (b) Decision tree on a trade-off between quantity and quality of life. (c) Resolution of a decision analysis. (d) Risk analysis. (e) Sensitivity analysis. In all cases, small squares label decision nodes, small circles label chance nodes, lines correspond to branches with treatment options (after decision nodes) or possible scenarios (after chance nodes) and final rectangles contain utility values.

Decision trees are made up of decision nodes, chance nodes, terminal nodes and branches connecting them. *Decision nodes* are marked by small squares from which *branches* originate corresponding to medical options among which to select the best. All mutually exclusive medical options reasonably conceivable by the clinician need to be exhaustively foreseen at each decision node. *Chance nodes* are marked by small circles from which branches linked to probabilities originate, corresponding to all mutually exclusive events which one can envision may take place. *Terminal nodes* are marked by rectangular boxes corresponding to final outcomes, which are linked to utilities, unless the decision tree is only probabilistic. A basic decision tree is sketched in Figure 1a: a decision node represents the alternative between treatment A and treatment B: each of them leads to a chance node representing the alternative between two possible outcomes, cure and no cure.

While it is the physician who must decide at the level of a decision node, it is the reality that decides at the level of a chance node. A physician can only convert this state of “uncertainty” into a state of “risk” by estimating *probabilities*, expressed by numbers between 0 to 1. In Figure 1a, cure after each option will be accompanied by a probability. Since envisaged consequences need to be exhaustive, the sum of all probabilities at each chance node will always be = 1. In the figure, branches departing from each chance node are just two, and thus, by definition, the probability associated to no cure will be equal to 1 - P cure.

In the end, after one or more layers of chance nodes (possibly, also of embedded decision nodes, if any), there will be a set of terminal nodes, corresponding to final outcomes. The number 0 or 1 - associated with how the patient under risk values an outcome - is called *utility* and is placed in the relevant terminal node. In Figure 1a, cure is associated with a utility of 1 and no cure with a utility of 0. If so, this would actually mean that the decision analysis will just provide an *expected probability* of cure.

However, there may be outcomes whose meaning to the patient are “intermediate”, in a sense, between the best and the worst quality of life: for example, being cured through treatments with long-term sequelae. In that case, the utility value of cure with sequelae may be assumed to lie somewhere between 1 and 0. A methodologically sound way of eliciting patient’s utility value is to establish which probability of the worst outcome, e.g. of death, he/she will be ready to accept in order to avoid sequelae. If the patient accepts a probability of dying of 0.1, then 1 - 0.1 = 0.9 will be his/her utility value for the intermediate outcome. In fact, he/she will be indifferent between the certainty of cure with sequelae (the intermediate outcome) on one side and a chance of 10% of dying and 90% of being cured without sequelae on the other side. This method is called “standard gamble”. In a sense, the utility will be elicited as a probability and will thus be suitable to be weighed by other probabilities in the decision tree. The final result of the decision analysis will then be an *expected utility*. Figure 1b represents a decision between a treatment resulting in removal of an organ (such as larynx, a limb, an eye, etc.) and another resulting in organ preservation, where probabilities of cure may be higher for the former and lower for the latter, thus implying a trade-off between quantity and quality of life.

A decision tree can be used under different perspectives. For example, in spite of placing patient’s utilities, one may put life years as outcomes in the terminal nodes. In this case, the final expected value will be *life expectancy*, since probabilities throughout the tree will be multiplied by life years. Life expectancy may also be weighed by utility values. For example, 10 years of life expectancy after an organ loss may be multiplied by a utility value of 0.9, giving rise to 9 Quality-Adjusted Life Years (QALYs). In case diminished quality of life affects only a portion of life expectancy, the utility value will be multiplied only by the relevant time interval and the product added to the rest of life years (i.e. multiplied by 1). Life expectancy and quality-adjusted life expectancy are meaningful under a population’s perspective, as matters in economic analyses, but are of little help under the perspective of the individual patient. What will matter to the individual patient will be how likely it is that, for example, he/she will be cured or not, or will have a major benefit, temporary though it may be, or not, rather than a median value statistically combining radically diverging outcomes across different patients.

A decision tree may be made up of several layers of chance (and possibly decision) nodes, leading to terminal nodes associated to utility values, as in Figure 1c. Folding back the tree implies calculating the expected value, i.e. the expected utility, of each chance node, from the end of the tree backwards to its root, as outlined in the figure. Finally, an expected utility will be associated to each option foreseen by the decision node at the root of the tree. *Maximizing expected utility* will mean choosing the option linked to the highest expected utility. If there are embedded decision nodes throughout the tree, at each of them the choice will correspond to their branch with the highest expected utility.

A decision tree can also be examined only probabilistically. Thereby, a *risk analysis* will be obtained by simply computing the sum of all path probabilities leading to the same outcome for each option, where all probabilities along a path are multiplied by each other from the root to the terminal nodes, as in Figure 1d. Thus, the tree will provide an *expected probability* of each outcome under each treatment option, and it will be up to patient’s judgement to select the option associated with the best risk profile. The analysis will incorporate the variety of outcomes, but will not foresee any utility values, because it will be the patient who will directly factor in his/her preferences when comparing options looking at the expected probabilities of outcomes.

Both probabilities and utilities will carry a degree of uncertainty. A *sensitivity analysis* may then show how the decision will be affected by changing the values of probabilities or utilities. In Figure 1e, the changing variable (in this case, a probability only affecting treatment B) is on the X axis, while the expected utility for the two options is on the Y axis. This is called a “one-way” sensitivity analysis. It will also serve as a threshold analysis, showing at which variable’s value the options would provide the same expected utility. “Two-way” sensitivity analyses are feasible as well. With proper software, one can also input several probability distributions and utility ranges throughout the decision tree, in spite of probability and utility point values. Then the software runs the decision analysis a high number of times, each time picking up a random value from probability distributions and utility ranges. Each time an option will turn out as the best, so that, finally, there will be a proportion of times when any option will have been better than the others. This will convey an idea of the stability of the decision given all the uncertainty inbuilt in the analysis. For example, one could be confident at a level of 70% about an option, if it will have turned out to be better in 70% of simulations. This is called a multivariate “Montecarlo” sensitivity analysis.^16^

The reader is referred elsewhere for details about MDA and further aspects of the method.^3-8^ Some tools of MDA are useful for economic decision analyses on the dual outcomes of costs and effectiveness, but not when MDA is envisaged for clinical use at the patient bedside: this is the case with *Markov analysis*, i.e. a simulation of the transition over time of a population of patients across different health states.^5,16-18^

### Decision tree templates for oncology

Hereafter we consider some typical scenarios of today’s treatment of cancer patients.^19^ For each of them we tentatively provide a basic decision tree template, which often will need to be enriched with additional nodes and branches to render challenging clinical decisions at the patient bedside. In all cases, utilities may be elicited from the individual patient to provide an expected utility analysis, or the tree may be kept only probabilistic, to provide the physician and the patient with a risk analysis for their shared decision-making process. All templates represent just one solution to structure a decision problem: other solutions will always exist.

We focus on cancer treatment and not on early cancer diagnosis or cancer risk testing, which clearly can be modelled as well in the single case, but which pose several issues going beyond the scope of this paper.

#### The quantity/quality of life trade-off in curable cancer disease

**Figure 2.**
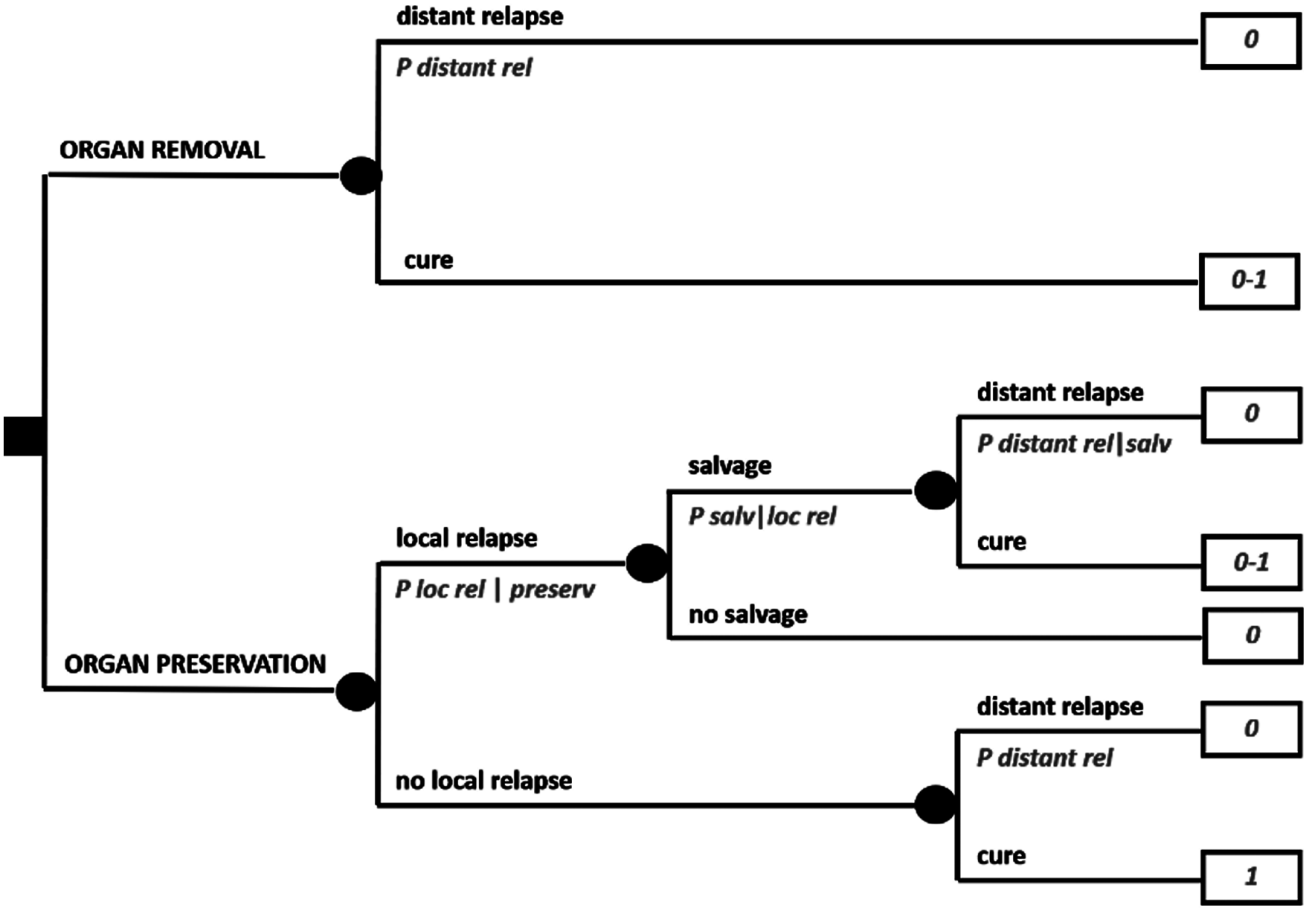
Expanded decision tree on a trade-off between quantity and quality of life.

Cancer treatments may imply serious side effects, as well as long-term toxicities and sequelae. Sometimes, for example, surgical treatments may lead to organ/function loss. Figure 1b (see above) formalizes this scenario. The same scenario is represented in a slightly more complex fashion in Figure 2, which, in addition, factors in the risk of a local relapse after conservative surgery, assuming that it can be rescued in a proportion of cases by organ removal, at that point with a possibly different systemic risk.

A typical clinical scenario like this, for example, may regard a sarcoma patient in whom the local lesion is fairly advanced, so that conservative surgery is technically feasible but there are reasons for believing that local relapse may be higher than the threshold usually accepted. What may be difficult to elicit from available studies are the probabilities of local relapse, since clinical presentations may be highly diverse. It may also be difficult to elicit the impact of a rescued local relapse on the systemic risk, which in principle could go from zero to a “new” baseline risk, or even a higher one due to some degree of dedifferentiation for example.

#### Adjuvant/neoadjuvant treatments in curable cancer disease

“Adjuvant” treatments are very common in today’s oncology. In solid cancers, surgery may often be followed by a medical therapy, with the aim of eradicating occult micrometastases and thus increase chances of cure. Sometimes, medical therapy is placed before surgery (“neoadjuvant” therapy), but the aim is the same. In general, any treatment modality may serve as an adjuvant to another.

**Figure 3.**
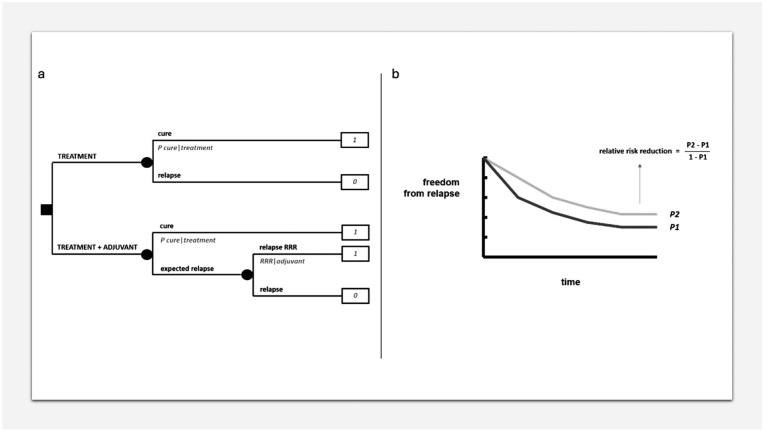
MDA in adjuvant/neoadjuvant setting. (a) Decision tree on an adjuvant therapy. (b) Relative risk reduction.

Figure 3a formalizes a decision between doing or not doing an adjuvant medical therapy after surgery. One option will be surgery alone, while the other will be adding adjuvant therapy to surgery. There will be a probability of cure with surgery alone and a relative reduction of the risk of relapse (1 - “P cure”) by doing adjuvant therapy. Assigning a utility of 1 to cure and 0 to relapse, the decision analysis will allow a simple risk analysis showing the absolute risk reduction. All this is conceptually important because it allows to obtain an absolute risk reduction in the individual patient depending on his/her specific baseline absolute risk. In fact, in the presence of a baseline absolute risk of 50% or 5%, the same relative risk reduction of 20%, will imply a completely different absolute risk reduction: 10% or 1%. Indeed, a mistake often made in patient information is to use relative, in spite of absolute, risk reductions.

The relative risk reduction can be calculated from a typical relapse-free survival curve of a clinical study comparing an adjuvant therapy with no adjuvant therapy, looking at the plateau of the two curves, as in Figure 3b. Of course, when exploiting such a relative risk reduction for different baseline risks, the assumption is that the same relative risk reduction will apply equally to all of them, which is far from a given and needs to be evaluated depending on the treatment and the disease.

On the other hand, as far as the baseline risk is concerned, this can be generally provided by prognostic nomograms, real-world case series, etc.

Of course, one could also incorporate a different utility value for the time spent on therapy, although usually the impact therefrom will be limited vis-à-vis chances of definitive cure.

#### Cytoreductive treatments

**Figure 4.**
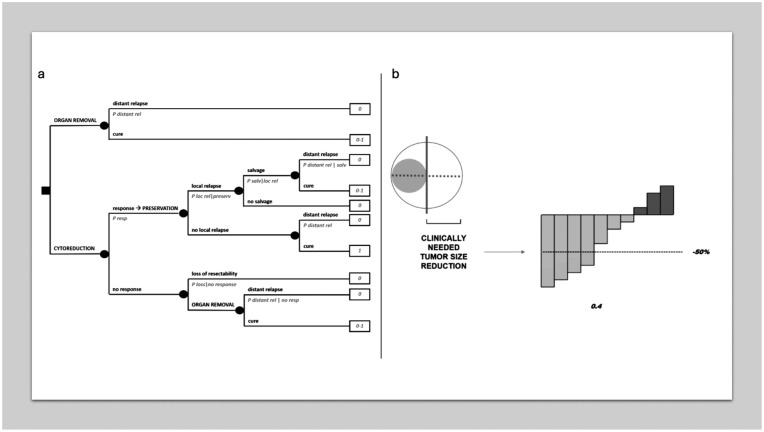
MDA in cytoreductive treatment. (a) Decision tree on cytoreduction for conversion to conservative treatment. (b) Clinically relevant threshold of a tumor response.

Sometimes a tumor is not amenable to potentially eradicating treatments, typically surgery, because of, for example, anatomical constraints. In these cases, a cytoreduction of a sufficient degree, as allowed by a medical therapy for example, can convert the tumor to a resectable state. Sometimes, the clinical choice may be about converting the tumor state not to surgical resectability as such, but to conservative surgery in spite of a mutilating one. This would lead to trying medical therapy. However, there may be fears that a surgical excision after cytoreduction entails lower chances of cure and/or that resectability is lost completely in case of progression of an insensitive tumor. Figure 4a provides a decision analysis modelling this clinical scenario, with a trade-off between quality of life (conservative treatment) and quantity of life (the extra-risk which may be in place, because of the conservative, in spite of a wider, excision after cytoreduction and/or the risk of interval progression). The model in Figure 4a assumes that there cannot be any local relapse after mutilating surgery, but of course this could be envisaged.

Probabilities of achieving a cytoreduction of a sufficient degree to allow a resection, or a conservative one, may be drawn from waterfall plots often provided by clinical studies on medical therapies in cancer. For example, in the individual case it may be assumed radiologically that a 50% regression in the main tumor diameter is needed to overcome surgical constraints to resectability, and a published waterfall plot may then be exploited to draw the probability of achieving such a 50% regression (see Figure 4b, in which it would be = 0.4). Of course, there will always be a random error in measurements provided by waterfall plots. Then, these will be often available in the advanced disease setting, and the assumption will be that local disease behaves like an advanced disease. However, it is important to stress that the clinically meaningful threshold will not necessarily correspond to arbitrary thresholds used in clinical research, e.g. the RECIST criteria and the like.^20,21^

More importantly, it may be difficult to quantify the risk of losing resectability in case of interval progression, although radiologically the surgeon may at least suppose that it is high, or low, or even nil. Obviously, the biological risk implied by surgical delay is much less easy to guess.

#### Active surveillance / watchful waiting

**Figure 5.**
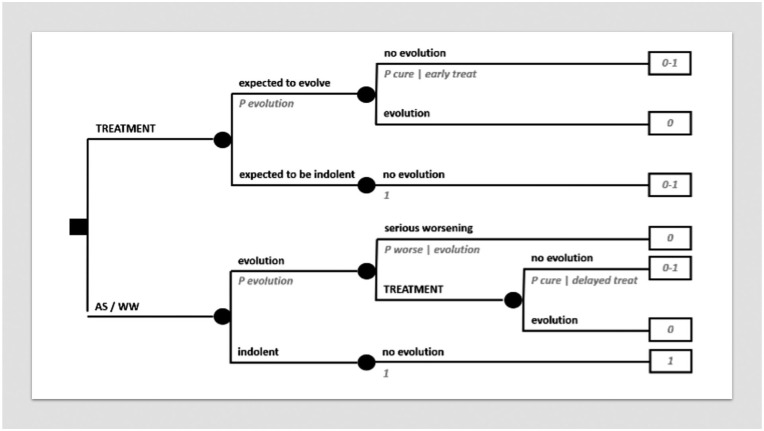
Decision tree on active surveillance / watchful waiting.

Some cancer presentations have an indolent natural history or at least are not due to evolve within patient’s expected life span. Thus, “active surveillance” or “watchful waiting” policies may be chosen in spite of any immediate treatment, which may carry significant side effects and/or sequelae. Prostatic cancer is a widely known example of this kind of scenario in oncology. Figure 5 models a decision between immediate treatment and active surveillance / watchful waiting, delaying treatment to when evolution, if any, becomes apparent. However, there may be the risk of some prognostic worsening if treatment is delayed, so that treatment becomes unfeasible at all. Thus, again, there may be a trade-off between quantity and quality of life.

#### The advanced disease

Advanced cancer disease may be curable in some settings, so that templates for localized curable disease may be applicable all the same.

**Figure 6.**
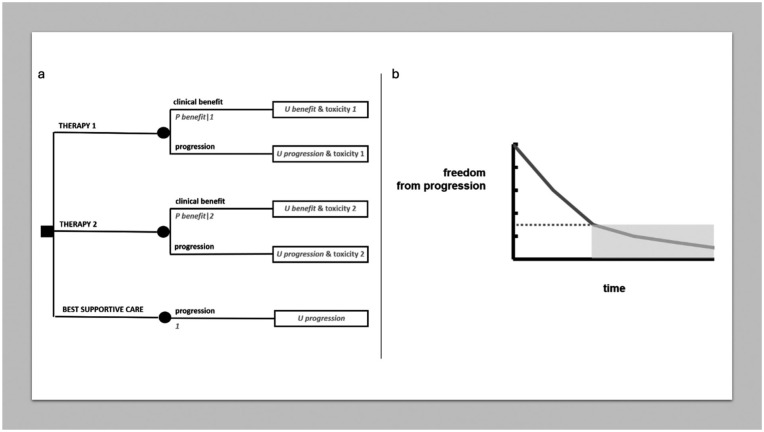
MDA in advanced disease. (a) Decision tree on advanced disease. (b) Breaking down freedom from progression into discrete time spans.

More often, advanced disease implies metastatic spread to several organs and the lack of any treatment reasonably able to result in significant chances of cure. However, medical therapy is generally available, even with the option of several lines of medical treatment, which may be used in a step wise manner. In these cases, some kind of tumor response leading to a freedom from progression of some duration is a reasonable clinical target. In medical literature, it is often questioned to which extent tumor response and progression-free survival correlate with overall survival, giving rise to a relevant magnitude of the clinical benefit.^22^ While this transcends the scope of this paper, often the problem may not be whether tumor response or absence of progression are clinically meaningful as such, but which is their duration. This said, a decision analysis as reproduced in Figure 6a may model a clinical decision in advanced cancer disease. A proportion of patients may be expected to gain some kind of what can be called a “clinical benefit” from treatment (often a medical therapy). In fact, using a median value of progression-free survival for all would fail to reflect the different experience of patients who actually “benefit” and of those who do not. Thus, the clinical benefit may be viewed as a tumor response of some degree, again not necessarily a tumor response meeting the threshold criteria used in clinical research, or it may be just an absence of progression. However, the clinician and the patient should agree, say, that in order to qualify for a clinically meaningful clinical benefit it should have a given duration. Thus, the tree simply makes a distinction between patients who “respond” in this sense and patients who do not. A choice may be to exploit freedom-from-progression curves, isolating the proportion of patients having an absence of progression for a meaningful time span, under the patient’s perspective, as in Figure 6b. This would dichotomize the progression-free survival curve, but of course the curve could also be broken down in more than two portions and be incorporated accordingly in the tree, i.e. with more than two branches, each with a probability and a time interval duration.

The third option in Figure 6a corresponds to a decision as to whether “to treat or not to treat” at all (always recalling that not to treat will actually mean “best supportive care”, i.e. a treatment as well), since such a choice may be in place in some advanced cancer disease settings, especially when several lines of medical therapy have already been exploited. However, the decision-making is conceptually the same as above.

#### Cancer follow-up

**Figure 7.**
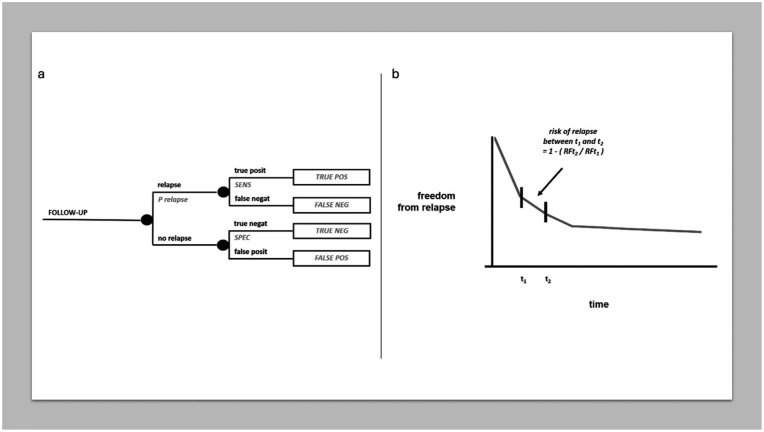
MDA in follow-up. (a) Chance node on cancer follow-up. (b) Risk of relapse in a follow-up window of time.

Although the follow-up of potentially cured cancer patients today focuses on the broad spectrum of patient “survivorship” (especially incorporating all what determines long-term quality of life), and the efficacy of an early diagnosis of systemic relapse has been questioned in some tumors, many cancer patients who received potentially curative treatment undergo some kind of “regular” follow-up in clinical practice. Of course, the benefit of regular follow-up will depend on the extent to which making an earlier diagnosis of relapse impacts prognosis, and this should be demonstrated, vis-a-vis the drawbacks of intense diagnostic testing (in terms of side effects like radiation exposure, false positives, psychological distress and the like). Assuming there is a benefit, one may wonder which is the probability of picking up a relapse within a given follow-up window. Figure 7a represents a chance node combining the probability of relapse and the sensitivity and specificity of the diagnostic exam. The risk of relapse can be drawn easily from a relapse-free survival curve by selecting the planned follow-up window, as shown in Figure 7b. This could help tailor the intensity of follow-up to individual risks and preferences, though within the scope, often undefined, of the potential effectiveness of an early diagnosis of relapse.

### Methodological difficulties and solutions

#### Probabilities

Some specific difficulties in eliciting probabilities at the patient bedside have already been mentioned.

A general difficulty in principle is that medical statistics has been developed following a frequentist paradigm. Conceptually, clinical trials do not provide probability distributions of efficacy. On the other hand, the Bayesian process for drawing such probability distributions from clinical trials is conceptually clear.^23^ However, it implies a degree of subjectivity, which may be viewed as a limiting factor of all Bayesian approaches. Authors believe that this could be a reasonable price to pay at the patient bedside, where probabilities are needed. As a matter of fact, probabilities are extensively inferred by clinicians in their everyday practice from data about survival (including relapse-free and progression-free survival), tumor response, toxicity and quality of life, as reported in published studies. However, this is done empirically, in the lack of formal processes to adjust these probabilities given the variability of published studies, and their uncertainty is hardly incorporated. A special difficulty has to do with the reliability of subgroup analyses in clinical studies, which are meant to test hypotheses in the “average patient”. On the other hand, these groups are crucial to elicit probabilities specifically adapting to the individual case. Possibly, attempts could be made to complement state-of-the-art instruments, like clinical practice guidelines, with evidence-based probability distributions based on some kind of consensus within the community of experts.

This said, decision analysis at the patient bedside inevitably requires resorting to “subjective” probabilities. Practically speaking, physicians are often afraid of estimating subjective probabilities, because they feel that this would be methodologically unsound. Paradoxically, they are less afraid of making any clinical decision than to assign probabilities throughout a “formal” decision tree. It may be simple to argue that guessing a probability is much less of a commitment than making a definitive clinical decision, and that the latter will in any case be based on some kind of probabilistic reasoning, so that making it explicit can only improve the whole process. Methodological research on how to translate the results of available clinical trials into subjective probability distributions at the patient bedside could help. Medical education could try to refine the cognitive abilities of medical students in estimating sound subjective probabilities based on evidence, for example by using penalty scores and the like.^24^ The clinician should also come to terms with an inevitable degree of approximation (such that estimations may well be made, for example, by intervals of 0.05, save for very low probabilities, etc.). By accepting this, a physician may become more comfortable with guessing subjective probabilities and using them in MDA at the patient bedside.

#### Utilities

Eliciting a utility value from the individual patient through the standard-gamble method is not difficult. However, no patient would accept or refuse, for example, an amputation just because of a decision analysis maximizing expected utility, individually though utilities were elicited. More practically, a probabilistic decision analysis can provide him/her with a risk analysis. Then, the good physician will carefully accompany that patient through a difficult decision, making it a truly shared clinical decision. An expected utility analysis may be useful to the clinician as a control of the consistency of the decision shared with the patient, but our impression is that a risk analysis will be generally more useful to the patient than any utility analysis.

This said, it is hard to overestimate the conceptual importance of the notion of utility, as a value estimated by the single patient under risk. The concept should be stressed in medical education. The individual subjectivity of utilities in medical decision-making also represents a limiting factor of utility assessments made in clinical studies, since they will only reflect the “average patient” of such studies. In the end, this is a limitation of quality-of-life research in general, as far as individual clinical decision-making is concerned.^25^

#### Ethical considerations on bedside MDA

MDA is a powerful tool for the physician to improve the quality of medical decisions. However, it is the physician that builds the decision model. In a sense, it formalizes his/her clinical judgement, which may be appropriate or wrong. This is why it is always important to refer to existing “state of the art” (i.e. to clinical practice guidelines and the like). Operationally and ethically, this assures that clinical decision-making remains within the scope of standard of care.

MDA is also a powerful tool to improve patient-physician shared decision-making. This requires a competent patient. In the case of low health literacy, or, even worse, cognitive impairment and the like, shared decision-making is obviously difficult. However, this is also true when MDA is not used.

#### The scope of MDA at the patient bedside

One should always be aware that MDA does not discover new evidence, unlike clinical trials. In other words, one should always avoid the temptation to use the combination of probabilities gained here and there as a substitute for missing knowledge. The model of adjuvant trials is a good example. One needs a clinical trial on the effectiveness of an adjuvant treatment, and only then will be able to modulate the absolute risk reduction across different patients with a different baseline risk (though paying attention to the assumptions which this implies). On the other hand, it is true that, in the lack of knowledge, a clinical decision in the individual patient must be made “now”, so that MDA can help in any case at the individual patient bedside.

While, particularly in the area of oncology, we live at a time of what is called precision, or personalized, medicine, one must acknowledge that we lack a convincing clinical method to implement it. Certainly, clinical trials may be undertaken in more and more specific molecular subgroups of patients. However, evidence-based medicine will always be affected by the problem of the “average patient” of clinical trials.^26^ In principle, MDA may be viewed as a clinical method to personalize our decisions based on available evidence. There may be an apparent loss in “precision” in all this (paradoxically), but this is a reasonable price to pay at the patient bedside.

**Figure 8.**
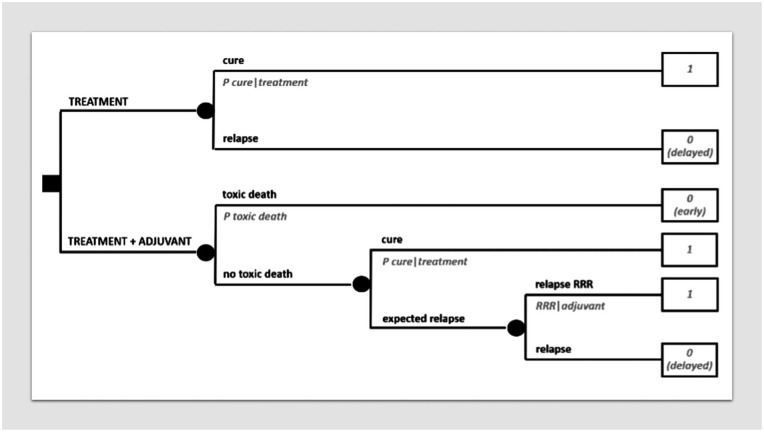
Decision tree on adjuvant therapy incorporating a severe toxic risk.

Obviously, the added value of MDA increases as long as the complexity of the decision increases. For example, the decision analysis on adjuvant therapies in Figure 3a underlies many tools often used every day by medical oncologists. However, such tools are available only for some cancers, generally not the rare ones. More important, any further variable pertaining to the single case would require a higher degree of personalization. For example, if a severe toxicity is expected from an adjuvant therapy in the single patient due to co-morbidities, it should be factored in. Figure 8 represents the same decision tree in Figure 3a with the inclusion of a potentially fatal toxicity (for obvious reasons, if it is an early death, it needs to be distinguished by risk analysis from delayed death, even when assigned the same utility of 0). This may clarify in principle how the basic templates provided in this paper can be enriched for those challenging decisions in which MDA is actually needed more, i.e. by adapting them to the variety of individual presentations. Although it is clear that MDA at the patient bedside will always entail a degree of simplification of patient’s complexity, its added value has to do with more or less complex decisions. It will be up to the clinician to model the decision problem to keep it as simple as possible while respecting its complexity. On the other hand, sometimes even a partial, non-quantitative, formalization of the decision problem by sketching a decision tree may substantially help the clinician to grasp the clinical problem and thereby to get straight to the final decision. In practice, however, this may make the difference between implementing, say, clinical practice guidelines prescribing to use adjuvant therapy and deciding not to use it in the single patient. In other words, MDA may lead the physician to rationally deviate from state of the art.

If formal MDA is not needed when the number of variables is low, it is true that some confidence with its method will be useful even when mentally processing simple decisions. Likely, a physician with some knowledge of the principles of MDA will be more appropriate also in his/her intuitive clinical decision-making at the patient bedside. In other words, MDA may play a crucial role in medical education.

This said, one cannot underestimate that employing MDA at the patient bedside requires considerable time and commitment. This is why we stress the educational value of MDA, since a physician trained in MDA may be more able to rapidly get to a shared medical decision even without formally using the tool. On the other hand, software packages are available for MDA, though primarily conceived for health economics.^27^ Actually, we believe that there would be room for user-friendly software for medical decision-making. The incorporation of a decision-analytic logic in “clinical decision support systems” would probably help make decision analysis more available to less methodologically-skilled physicians also.

In conclusion, MDA is a rigorous, although simple, method to personalize medical decision-making at the patient bedside. Obviously, it does not build new evidence, but it may help make the most of available evidence. Again, it is just a tool to improve medical decision-making. It has a great potential in medical education to teach the method of clinical decision-making. It could also be useful in many medical decisions implying some complexity and/or a trade-off between quality and quantity of life. Hopefully, in the near future its use at the patient bedside could be fostered by means of clinical decision support systems, as long as these may choose to incorporate a decision-analytic, not just an algorithmic, logic.
